# Supplementary material for: Paternal indifference and neglect in early life and creativity: Exploring the moderating role of TPH1 genotype and offspring gender
Source: PLoS One. 2020 Jul 29;15(7):e0221383. doi: 10.1371/journal.pone.0221383 (PMC7390402; doi:10.1371/journal.pone.0221383)
Supplement: Attachment — Submitted filename: Response to Reviewers.docx [file pone.0221383.s002.docx]

Dear editor and reviewers,

Thank you for your letter and for the reviewers’ comments concerning our manuscriptentitled “Paternal Indifference and neglect in early life and Creativity: Exploring the Moderating Role of TPH1 genotype and Offspring's Gender” (ID: PONE-D-19-21789). Those commentsare all valuable and very helpful for revising amd improving our paper,as well as the important guiding significanceto our researches. We have studied comments carefully and have made correction which we hope meet with approval. Enclosed is a revised manuscript according to the comments of the reviewers. Revised portion are marked in red in the paper. The main correction in the paper and the responds to the reviewers’ comments are listed below:

Responds to the Journal Requirements:

1. **Response to comment:** Please ensure that your manuscript meets PLOS ONE's style requirements, including those for file naming.

**Reply:** We have revised the format of our manuscript to meet PLOS ONE's style requirements.

2. **Response to comment:** Please outline how gender was determined in your Methods section.

**Reply:** We have outlined how gender was determined in our revised Methods section(Page 10, Line 191).

3. **Response to comment:** We note that you have indicated that data from this study are available upon request. PLOS only allows data to be available upon request if there are legal or ethical restrictions on sharing data publicly. For more information on unacceptable data access restrictions, please see http://journals.plos.org/plosone/s/data-availability#loc-unacceptable-data-access-restrictions.

In your revised cover letter, please address the following prompts:

a) If there are ethical or legal restrictions on sharing a de-identified data set, please explain them in detail (e.g., data contain potentially sensitive information, data are owned by a third-party organization, etc.) and who has imposed them (e.g., an ethics committee). Please also provide contact information for a data access committee, ethics committee, or other institutional body to which data requests may be sent.

b) If there are no restrictions, please upload the minimal anonymized data set necessary to replicate your study findings as either Supporting Information files or to a stable, public repository and provide us with the relevant URLs, DOIs, or accession numbers. For a list of acceptable repositories, please see http://journals.plos.org/plosone/s/data-availability#loc-recommended-repositories.

We will update your Data Availability statement on your behalf to reflect the information you provide.

**Reply:** We sincerely apologize for we misunderstood the meaning of "available upon request". There are no restrictions, and we have upload the minimal anonymized data set as Supporting Information files (S1_DATA).

Responds to the reviewers’ comments:

Reviewer #1:

1. **Response to comment:** The theoretical background of this study seems to be weak and unconvincing. Why, for instance, should parental indifference & neglect be associated with creativity? And the paper examined the TPH1 rs623580 × offspring’s gender interactions on creativity, but it seems lacked the theoretical or empirical evidence in introduction part.

**Reply:** Thank you for your helpful advice. We have revised our introduction part to better illustrate the theoretical and empirical background of this study. We have added a paragraph subtitled “Role of paternal indifference & neglect” (Page 6, Line 94) to specify the reasons for our study of the relationship between parental indifference & neglect and creativity. Secondly, we have added three paragraphs to specify the reasons for our study examining the paternal indifference & neglect × offspring gender interaction(Page 8, Line 152), the TPH1 rs623580 × offspring gender interaction (Page 9, Line 165), and the three-way interaction(Page 9, Line 174).

2. **Response to comment:** The Parental Bonding Instrument consist of care, indifference & neglect, overprotection and autonomy, but why only care and indifference & neglect was used in the present paper. Were overprotection and autonomy related with creativity?

**Reply:** Thank you for your helpful advice. The reason why we only used care and indifference & neglect in this study is that the role of paternal indifference & neglect in creativity is what we are interested in. Previous research has reported that the two subscales parental care and indifference & neglect are highly related. Therefore, we also used care in the present paper. Although overprotection and autonomy have been reported to be relate to creativity, they are not the issues to be investigated in this study. Therefore, overprotection and autonomy were not used in the present paper.

3. **Response to comment:** Whether mean centering was performed before moderation analysis?

**Reply:** Thank you for your helpful advice. Mean centering was performed before moderation analysis. We have added the description of mean centering in our revised manuscript(Page 13, Line 257).

4. **Response to comment:** Were scores of the creativity (fluency, flexibility and originality) normally distributed? If not, how was this accounted for in the statistical model that calls for normality?

**Reply:** Thank you for your helpful advice. The scores of the creativity (fluency, flexibility and originality) were not normally distributed.

In the initial data analysis, we found that the scores of the creativity (fluency, flexibility and originality) were not normally distributed, So we corrected the data sets to follow normal distribution, respectively. Then, we conducted two data analyses with the original data sets as the dependent variables and the corrected data sets as the dependent variables. It was found that the results with the original data sets as the dependent variables were similar to those with the corrected data sets as the dependent variables (The results of data analysis using corrected data as the dependent variable are as follows. Table C1, C2). Since the original scores of creativity are generally used in the existing literature[1, 2], we reported the results of using the original scores as the dependent variables in the original manuscript, but did not report the results of using normalized corrected data sets.

[1] Zabelina D L , Colzato L , Beeman M , et al. Dopamine and the Creative Mind: Individual Differences in Creativity Are Predicted by Interactions between Dopamine Genes DAT and COMT. PLOS ONE, 2016, 11(1).

[2] Runco MA, Noble EP, Reiter-Palmon R, Acar S, Ritchie T, Yurkovich JM. The Genetic Basis of Creativity and Ideational Fluency. Creativity Research Journal. 2011;23(4):376-380. doi: 10.1080/10400419.2011.621859. PubMed PMID: WOS:000299566100010.

Table C1 Hierarchical linear regression analysis testing the effects of Paternal indifference & neglect, TPH genotype and their interaction on corrected creativity scores.

| **Variables** | **Model 1** | **Model 2** | **Model 3** | **Model 4** | **Model 5** | **Model 6** | **Model 7** | **Model 8** | **Model 9** |
| --- | --- | --- | --- | --- | --- | --- | --- | --- | --- |
|  | **Fluency** | **Fluency** | **Fluency** | **Originality** | **Originality** | **Originality** | **Flexibility** | **Flexibility** | **Flexibility** |
|  | ***β*** | ***β*** | ***β*** | ***β*** | ***β*** | ***β*** | ***β*** | ***β*** | ***β*** |
| **Age** | -.005 | -.007 | -.015 | .011 | .009 | .002 | -.020 | -.022 | -.031 |
| **PC** | .127^**^ | .107 | .108 | .119^**^ | .105 | .105 | .108^*^ | .106 | .106 |
| **PI** |  | -.026 | .108 |  | -.018 | .094 |  | -.002 | .138 |
| **rs623580** |  | -.066 | -.066 |  | -.049 | -.049 |  | -.070 | -.070 |
| **PI × rs623580** |  |  | -.165^*^ |  |  | -.139^†^ |  |  | -.173^*^ |
| **F** | 4.458^*^ | 2.847^*^ | 3.343^**^ | 3.809^*^ | 2.240 | 2.547^*^ | 3.418^*^ | 2.372^*^ | 3.074^**^ |
| **R^2^** | .016 | .021 | .030 | .014 | .017 | .023 | .013 | .017 | .028 |
| ***△*R^2^** | .013 | .014 | .021 | .010 | .009 | .014 | .009 | .010 | .019 |

Note. Male = 1, Female = 0; PC= Paternal care, PI= Paternal indifference & neglect; ^†^*p = .05,*^*^*p < .05*, ^**^*p < .01*

Table C2 Hierarchical linear regression analysis testing the effects of TPH genotype, offspring gender and their interaction on corrected creativity scores.

| **Variables** | **Model 1** | **Model 2** | **Model 3** | **Model 4** | **Model 5** | **Model 6** | **Model 7** | **Model 8** | **Model 9** |
| --- | --- | --- | --- | --- | --- | --- | --- | --- | --- |
|  | **Fluency** | **Fluency** | **Fluency** | **Originality** | **Originality** | **Originality** | **Flexibility** | **Flexibility** | **Flexibility** |
|  | ***β*** | ***β*** | ***β*** | ***β*** | ***β*** | ***β*** | ***β*** | ***β*** | ***β*** |
| **Age** | -.018 | .012 | .008 | -.001 | .021 | .018 | -.031 | -.011 | -.016 |
| **rs623580** |  | -.087^*^ | -.040 |  | -.065 | -.027 |  | -.086^*^ | -.018 |
| **Offspring gender** |  | -.318^***^ | -.235^**^ |  | -.239^***^ | -.172^*^ |  | -.228^***^ | -.108 |
| **rs623580×Offspring gender** |  |  | -.112 |  |  | -.090 |  |  | -.162^*^ |
| **F** | .176 | 20.181^***^ | 16.252^***^ | .001 | 11.109^***^ | 8.703^***^ | .523 | 10.818^***^ | 9.365^***^ |
| **R^2^** | .000 | .105 | .109 | .000 | .059 | .061 | .001 | .057 | .066 |
| ***△*R^2^** | -.002 | .100 | .102 | -.002 | .053 | .054 | .000 | .052 | .059 |

Note. Male = 1, Female = 0; PC= Paternal care, PI= Paternal indifference & neglect; ^*^*p* *< .05*, ^**^*p* *< .01*, ^***^*p* *< .001*

Special thanks to you for your good comments.

Reviewer #2:

Major concerns:

1. **Response to comment:** The overall structure of Introduction is slightly confusing and there is a lack of clarity. For example, the part subtitled “TPH1 rs623580 and creativity” in Introduction was actually about the relation between paternal indifference & neglect in early life and creativity in youth may be moderated by genetic variants.

**Reply:** Thank you for your helpful advice. We have revised the structure of Introduction. We have added a paragraph subtitled “Role of paternal indifference & neglect” (Page 6, Line 94) to specify the reasons for our study of the relationship between parental indifference & neglect and creativity. Secondly, we have added three paragraphs to specify the reasons for our study examining the paternal indifference & neglect × offspring gender interaction(Page 8, Line 152), the TPH1 rs623580 × offspring gender interaction (Page 9, Line 165), and the three-way interaction(Page 9, Line 174).

2. **Response to comment:** It’s better going deeper into Discussion. It is not clear what these findings mean for our further understanding of the development of creativity as well as the cultivation of creativity. It should be discussed further.

**Reply:** Thank you for your helpful advice. We have revised it according to your comments. We have added a paragraph to discuss findings in present study mean for further understanding of the cultivation of creativity (Page 23, Line 424).

3. **Response to comment:** Both “moderation” and “interaction effects” are very much similar to each other. Mathematically, they both can be modelled by using product term in the regression equation. Researchers often use the two terms as synonyms but there is a thin line between interaction and moderation. When we say X and Z interact in their effects on an outcome variable Y, and there is no real distinction between the role of X and the role of Z. They are both considered predictor variables. Then we identify this effect as interaction effect. While, in case we have clear distinction between the predictor and moderator variables (on the basis of theory) and we are interested to see the impact of predictor on response (affected by moderator), then this effect is known as moderation effect. The authors should carefully choose the term which is more suitable to answer their research question.

**Reply:** Thank you for your helpful advice. According to the hypothesis of this study, Paternal Indifference and neglect is the predictor variable, and TPH1 genotype and Offspring's Gender are both moderator variables. Thus, in the revised Introduction and Discussion section, we have consistently modified the interaction to a moderation effect according to our hypothesis. Since we used the research method of G×E INTERACTION, we still retained the interaction in the results section.

4. **Response to comment:** The Results reported the findings of the interaction effect of paternal indifference & neglect and TPH1 rs623580 as well as the interaction effect of TPH1 rs623580 and offspring’s gender on each outcome variable (fluency, flexibility, and originality), but the interaction effect of paternal indifference & neglect and TPH1 rs623580 on fluency, flexibility, and originality didn’t be introduced in detail in Abstract but stated simply “creativity”. Moreover, if there was a different pattern of results for each outcome variable (fluency, flexibility, and originality), it had better be discussed properly in Discussion.

**Reply:** Thank you for your helpful advice. we have revised the Abstract section according to your comments, and we have added the interaction effect of paternal indifference & neglect and TPH1 rs623580 on fluency, flexibility, and originality. Because the results for each outcome variable (fluency, flexibility, and originality) present the same pattern, thus we did not discuss them separately in the discussion.

**Response to comment:** Related to this is another issue: in METHODS, the authors did not introduce how they obtained creativity score, although they explained how they obtained fluency, flexibility, and originality scores.

**Reply:** Thank you for your helpful advice. We are sorry that this part was not clear in the original manuscript. According to Guilford (1967), creativity refers to embodiment of a thought consisting of three characteristics: fluency, flexibility, and originality. Thus, we used these three characteristics as outcome variable in this study, and we did not obtain creativity score.

5. **Response to comment:** Previous studies indicated that gender difference might be attributed to the interaction effect of genetic and environmental factors on creativity. What is the relationship between the findings in the present study and in previous studies? The cause of non-significant three-way interaction effect on three outcome variables was not discussed in Discussion.

**Reply:** Thank you for your helpful advice. We have added a paragraph to discuss the non-significant three-way interaction effect on three outcome variables (Page 23, Line 424) in our revised manuscript. In this paragraph, we also have tried to discuss relationship between the findings in the present study and in previous studies.

Minor concerns：

1. **Response to comment:** The necessary information on Measures in METHODS should be included. For example, “Originality score was calculated by counting the number of unusual ideas given by less the 5% of the sample.” Please described in much more detail about “the sample”. Similarly, “To score flexibility, a category list was first generated for each item, and the flexibility score was the number of different categories used in one participant’s ideas.” Please described in much more detail about “a category list”.

**Reply:** Thank you for your helpful advice. We have revised Methods section to clarify the criteria by which we evaluate originality scores, and We have added some examples to describe the “a category list” we used to evaluate flexibility scores

2. **Response to comment:** It is noted that the manuscript needs careful editing. needs to be. Some sentences are awkward.

For example:

Syntax errors：“One possible explanation is that the influence of parental indifference & neglect to children may be differ between mother and father.”

“It is postulated that paternal indifference & neglect in early life would be negatively predict creativity in youth.”

“The findings of the present study contribute to further understanding the role of genetic factors in the pathways that how the early life family environment shapes creativity in adulthood.”

**Reply:** Thank you for your helpful advice, and we sincerely apologize for the Syntax errors in our previous manuscript. We have thoroughly and completely revised the entire manuscript. And we have made careful amendments to the questions raised in the manuscript.

These comments are quite helpful, and I revised my paper point-by-point. Thank you and the review again for your help!

**Response to comment:** While revising your submission, please upload your figure files to the Preflight Analysis and Conversion Engine (PACE) digital diagnostic tool, https://pacev2.apexcovantage.com/. PACE helps ensure that figures meet PLOS requirements. To use PACE, you must first register as a user. Registration is free. Then, login and navigate to the UPLOAD tab, where you will find detailed instructions on how to use the tool. If you encounter any issues or have any questions when using PACE, please email us at figures@plos.org. Please note that Supporting Information files do not need this step.

**Reply:** Thank you for your helpful advice. We have uploaded our figures files to the PACE digital diagnostic tool. Enclosed is the PACE Corrected figures.

Because some problems with the e-mail provided in the original manuscript, we have now changed the email address to [zhangjinghuan@126.com](mailto:zhangjinghuan@126.com)

We tried our best to improve the manuscript and made some changes in the manuscript. These will not influence the content and framework of the paper. And here we did not list the changes but marked in red in revised paper.

We appreciate for reviewer’s warm work earnestly, and hope that the correction will meet with approval.
