# Supplementary material for: Paternal indifference and neglect in early life and creativity: Exploring the moderating role of TPH1 genotype and offspring gender
Source: PLoS One. 2020 Jul 29;15(7):e0221383. doi: 10.1371/journal.pone.0221383 (PMC7390402; doi:10.1371/journal.pone.0221383)
Supplement: Attachment — Submitted filename: Response to Reviewers.docx [file pone.0221383.s003.docx]

Dear editor and reviewers,

Thank you for your letter and for the reviewers’ comments concerning our manuscript entitled “Paternal Indifference and neglect in early life and Creativity: Exploring the Moderating Role of TPH1 genotype and Offspring Gender” (ID: PONE-D-19-21789R1). Those comments are all valuable and very helpful for revising and improving our paper. We have studied comments carefully and have made correction which we hope meet with approval. Enclosed is a revised manuscript according to the comments of the reviewers. Revised portion are marked in red in the paper. The main correction in the paper and the responds to the reviewers’ comments are listed below:

Responds to the reviewers’ comments:

Reviewer #2:

1. **Response to comment:** The manuscript improved and some parts were clarified. The revised version is more well-organized than the previous one. However, the statement “Findings from the current study suggested that the A allele of TPH1 (rs623580) might be a risk allele for creativity, and the long-term negative influence of paternal indifference & neglect in early life on individuals’ creativity in youth depending on TPH1 genotype.” in Abstract is not prudent. One should be cautious in drawing general conclusions from the specific results.

**Reply:** Thank you for your helpful advice. We have rewritten the last sentence of our Abstract section according to your comments (Page 2, Line 23). We summarized the results of this study, and tried to avoid drawing inappropriate general conclusions.

2. **Response to comment:** I suggest the authors add the accurate references to the Divergent Thinking Test scoring methods for obtaining fluency, flexibility, and originality scores. Moreover, the scoring methods was still not described sufficiently. Given the relatively low Cronbach’s alpha for flexibility, more detailed information about the scoring methods should be provided.

**Reply:** Thank you for your helpful advice. We have revised Methods section according to your comments. Fristly, we added accurate references to the Divergent Thinking Test scoring methods for obtaining fluency, flexibility, and originality scores(Page 12, Line 225, Line 235). Secondly, we revised sentence whcih described the method for obtaining originality scores to further clarify the criteria we used (Page 12, Line 227). Moreover, we added sentences to describe the process we used to evaluate flexibility scores in detail, and we also added examples to describe the “a category list” (Page 12, Line 228).

3. **Response to comment:** Please double check the tense issue in Introduction and Discussion.

**Reply:** Thank you for your helpful advice. We have thoroughly and completely revised the entire manuscript. And we have made careful amendments to the questions raised in the manuscript.

We tried our best to improve the manuscript and made some changes in the manuscript. These will not influence the content and framework of the paper. And here we did not list the changes but marked in red in revised paper.

We appreciate for reviewer’s warm work earnestly, and hope that the correction will meet with approval.
